# Regulation of Connexin Gap Junctions and Hemichannels by Calcium and Calcium Binding Protein Calmodulin

**DOI:** 10.3390/ijms21218194

**Published:** 2020-11-02

**Authors:** Zhengping Hu, Manuel A. Riquelme, Sumin Gu, Jean X. Jiang

**Affiliations:** 1 Schepens Eye Research Institute of Massachusetts Eye and Ear, Boston, MA 02114, USA; Zhengping_Hu@MEEI.HARVARD.EDU; 2 Department of Ophthalmology, Harvard Medical School, Boston, MA 02114, USA; 3 Departments of Biochemistry and Structural Biology, University of Texas Health Science Center, San Antonio, TX 78229, USA; Riquelme@uthscsa.edu (M.A.R.); gus@uthscsa.edu (S.G.)

**Keywords:** connexin, calcium, calmodulin

## Abstract

Connexins are the structural components of gap junctions and hemichannels that mediate the communication and exchange of small molecules between cells, and between the intracellular and extracellular environment, respectively. Connexin (Cx) 46 is predominately expressed in lens fiber cells, where they function in maintaining the homeostasis and transparency of the lens. Cx46 mutations are associated with impairment of channel function, which results in the development of congenital cataracts. Cx46 gap junctions and hemichannels are closely regulated by multiple mechanisms. Key regulators of Cx46 channel function include Ca^2+^ and calmodulin (CaM). Ca^2+^ plays an essential role in lens homeostasis, and its dysregulation causes cataracts. Ca^2+^ associated CaM is a well-established inhibitor of gap junction coupling. Recent studies suggest that elevated intracellular Ca^2+^ activates Cx hemichannels in lens fiber cells and Cx46 directly interacts with CaM. A Cx46 site mutation (Cx46-G143R), which is associated with congenital Coppock cataracts, shows an increased Cx46-CaM interaction and this interaction is insensitive to Ca^2+^, given that depletion of Ca^2+^ reduces the interaction between CaM and wild-type Cx46. Moreover, inhibition of CaM function greatly reduces the hemichannel activity in the Cx46 G143R mutant. These research findings suggest a new regulatory mechanism by which enhanced association of Cx46 with CaM leads to the increase in hemichannel activity and dysregulation may lead to cataract development. In this review, we will first discuss the involvement of Ca^2+^/CaM in lens homeostasis and pathology, and follow by providing a general overview of Ca^2+^/CaM in the regulation of Cx46 gap junctions. We discuss the most recent studies concerning the molecular mechanism of Ca^2+^/CaM in regulating Cx46 hemichannels. Finally, we will offer perspectives of the impacts of Ca^2+^/CaM and dysregulation on Cx46 channels and vice versa.

## 1. Lens and Connexins

The lens is an avascular transparent organ suspended between the anterior chamber and the back of the eye. It focuses and transmits light to the retina. Due to the lack of direct blood supply, the lens obtains oxygen and nutrients primarily from the aqueous humor, and vasculature in the iris and retina [1]. Lens anatomy consists of an anterior capsule, a single layer of epithelial cells beneath the capsule, and the bulky lens fibers. The lens fiber cells, making up the bulk of the lens, are under different stages of cell differentiation. These lens fibers originate from epithelial cells at the lens equator and undergo extensive elongation and differentiation with the most mature lens fiber cells located in the lens nucleus [2]. Lens homeostasis is vital for its transparency, the dysregulation of which leads to cataracts, i.e., lens opacity, which is estimated to be the cause of blindness in 50% of people with the condition worldwide, making it the leading cause of blindness [3]. 

Gap junction channels are the pathways through which lens fiber cells communicate with each other and to cells at the lens surface [4]. These channels pass small molecules (<1.2 kDa) like ions, second messengers, and small metabolites between the lens cells [5]. Hemichannels, also called connexons, are composed of six connexin molecules. Connexins (Cx) are a family of structurally related transmembrane proteins with 21 identified members in human. Each connexin protein has four transmembrane domains, two extracellular loop domains, an intracellular loop domain, and N- and C-terminal domains. The intracellular loop and C-terminal domains are highly diverse among connexin subtypes [6]. Three connexins present in the lens are Cx43, Cx46, and Cx50, of which Cx46 and Cx50 are predominantly expressed in the lens fiber cells [7]. Epithelial cells predominantly express Cx43 and Cx50, while Cx50 and Cx46 are present in bulk of lens fiber cells. Mature lens fiber cells dominantly express Cx46 and Cx50 [8].

The lens gap junction channels form an internal circulation system that helps maintain cellular homeostasis in the lens. This circulation is coupled with fluid movement that allows ions and nutrients to flow into the center of the lens and metabolic waste to move out to superficial cells [9]. This process compensates the lack of vasculature within the lens. Ions such as Na^+^ and Ca^2+^ (rich in the extracellular environment) enter the lens circulation from the anterior and posterior poles, then move to the center through relatively high resistance extracellular spaces [9,10]. The driving forces are generated by an asymmetrical distribution of the Na^+^/K^+^-ATPases and Ca^2+^-ATPase that actively transport Na^+^ and Ca^2+^ ions [11]. These pumps that are in the surface of epithelial cells generate a low intracellular Na^+^ and Ca^2+^ concentration and establish an electrochemical gradient. The ions are driven into fiber cells by their transmembrane equilibrium potential. This energy is used for cell signaling and transport of extracellular nutrients, including glucose, amino acids, and peptides [12,13,14]. To maintain the Na^+^/Ca^2+^ gradient lens, fibers depend on passive diffusion through low resistant intercellular pathways created by gap junction channels [9,15,16].

The movement of ions along with the transmembrane fluxes create transmembrane osmotic gradients, coupling the movement of fluid to facilitate the efflux of ions from the interior of the lens, as well as the diffusion of nutrients, like glutathione from the outer cortex to the lens nucleus [17]. Glutathione is the principal antioxidant in the lens and maintains the oxidative homeostasis of lens, and Cx46 gap junctions transport glutathione to the center of the lens [17]. Lens fiber gap junction channels play a significant role in lens physiology and hemostasis. Any interruption in lens microcirculation will lead to cataract formation [5]. Therefore, maintaining the steep concentration gradient is a key factor in the transparency of the lens [18,19]. Indeed, the importance of connexin functions has been evident in single and double Cx knock out models. The absence of Cx46, Cx50, or mutations that block channel activity alters lens microcirculation and reduces metabolic homeostasis of the lens leading to congenital cataracts [20].

Albeit rich presence of gap junction structures in lens fiber cells, their presence between lens fiber and epithelial cells remains controversial [21,22] and thus far no evidence supports metabolic coupling through gap junction channels between fiber and epithelial cells. However, there is electrical coupling between the cells that allows for a unidirectional flow of ions and fluorescent molecules from fiber to epithelial cells [21,22,23], although the mechanism of this phenomenon remains obscure.

In addition to gap junction channels, the presence of lens Cx hemichannels has been postulated for years [20]. It has been shown that lens cell electrical properties respond to conditions that increase Cx hemichannel activity, such as low extracellular Ca^2+^, and this response is inhibited by Cx channel pharmacologic blockers [20]. The presence of lens hemichannels has been confirmed in isolated fibers cells using whole cells patch clamp and dye uptake assays [24]. After removal of divalent cations from the extracellular media, isolated fibers increase currents through the plasma membrane, increase propidium iodide and 4′-6-diamidino-2-phenylindole (DAPI) uptake. This current flow is inhibited by gap junction channel blockers including Gd^3+^, flufenamic acid, and octanol. Furthermore, current flow is absent in lens fibers without Cx46 expression. Fibers lacking Cx50 maintain current and dye uptake, suggesting that Cx46 hemichannels may be functional in vivo [24]. On the other side, the presence of functional Cx50 hemichannels has been shown in differentiated lens epithelial primary cultures system. Functional Cx50 hemichannels protect the cells against oxidative stress, providing an influx pathway for glutathione, which is rich in the extracellular vitreous humor and extracellular environment of the lens fiber [14,25].

## 2. Calcium and Calmodulin in Regulation of Lens Homeostasis and Pathology

Divalent calcium ions are involved in a multitude of cellular functions as an intracellular messenger and mediator within lens cells. To achieve this role, as a signaling molecule, intracellular Ca^2+^ concentration ([Ca^2+^]_i_) is strictly regulated to avoid the adverse effects of Ca^2+^ overload. The Ca^2+^ concentration in human aqueous humor is estimated at 1.34 mM. Human lens fibers [Ca^2+^]_i_ during resting condition is relatively high, around 9 μM, several times higher compared with lens epithelial cells that are around 80 nM [26]. Experimental measurements made in mouse lens show uneven [Ca^2+^]_i_ across different regions of the lens. Mature lens fibers have higher [Ca^2+^]_i_ close to 500 nM, and this concentration progressively decreases to roughly half in outer fibers. Ca^2+^ pumps that are enriched in the equatorial region of the lens drive the flow of water and ions. This microcirculation allows the distribution of nutrients and the removal of metabolic waste in the lens [9].

The Na^+^/Ca^2+^ exchanger and the plasma membrane Ca^2+^-ATPase are two major mechanisms involved in maintaining the Ca^2+^ concentration in the lens. Since the cell membranes have some basal permeability to Ca^2+^, continuous membrane transport of Ca^2+^ out of cells by these two pumps maintain the low cytoplasmic Ca^2+^ levels. The Ca^2+^-ATPase pump, which is highly concentrated at the lens equator region, mediates the intracellular flux of Na^+^ out of the lens [27]. Only lens epithelial cells express Ca^2+^-ATPase pumps; therefore, the removal of cytoplasmic Ca^2+^ in the lens fiber cells relies on the passive diffusion of Ca^2+^ through low resistant intercellular channels [15,28]. The maintenance and regulation of Ca^2+^ in the lens are more complicated due to its unique structural and physiological properties [15]. Evidence shows that lens membrane permeability increases in cataracts [29], leading to an elevated total lens Ca^2+^ level, and decreased Ca^2+^-ATPase activity [30,31]. Increased extracellular Ca^2+^ promotes the clustering and crystallization of highly organized gap junction channels in the cortical lens fibers. In parallel, the activation of calpain 3 results in proteolysis of spectrin, crystallins, and other critical substrates, which contributes to an opaque lens [32]. Gao et al. show that Cx46fs380 mutation disrupts lens internal circulation and leads to increased Na^+^ and Ca^2+^ concentrations, decreased epithelial Na^+^-K^+^-ATPase activity, and precipitation of Ca^2+^ in the lens [16]. The lack of Cx46 expression leads to severe nuclear cataracts, with [Ca^2+^]_i_ ranging between 0.7 to 1.5 μM, which is high enough to activate proteases and induce protein aggregation [15]. This experimental evidence suggests that the Ca^2+^ internal circulation is critical to lens homeostasis and transparency.

Calmodulin (CaM), a modulator in a wide variety of cellular processes, is one of the most important versatile Ca^2+^ signaling proteins in the cytoplasm of cells. CaM antagonists induce changes in lens permeability and transparency [33]. CaM is made up of 148 amino acid residues with two globular domains connected by a flexible linker. Each domain can bind two Ca^2+^ cations in its E-F hand motif, thus CaM can bind up to four Ca^2+^ cations [34]. Upon binding to Ca^2+^, the helix-loop-helix of CaM undergoes a conformational change, which couples with the flexibility of the protein, and allows the interaction between a wide variety of proteins as a Ca^2+^/CaM complex [35]. One of the essential functions of the Ca^2+^/CaM complex is to activate Ca^2+^ pumps on the plasma membrane and endoplasmic reticulum (ER), thus maintaining the Ca^2+^ levels in the cytoplasm and ER, and regulating downstream responses [36]. CaM antagonists can inhibit lens Ca^2+^-ATPase activity and lead to an accumulation of Ca^2+^ in the cytoplasm of lens fibers, thus inducing protein denaturation followed by lens opacification [37]. Aquaporin 0 (AQP0) is highly expressed in the lens fiber cells, and helps regulate water permeability in a Ca^2+^-dependent manner [38,39]. CaM is also necessary for Ca^2+^-dependent reduction of AQP0 channel permeability [40]. CaM regulates the conformational changes of AQP0 channels [41] by binding the positively charged C-terminal domain.

## 3. Ca^2+^ and Calmodulin in Regulation of Cx Formed Gap Junction Channels

It is well known that Ca^2+^ affects gap junction structure and cell–cell coupling [42]. Loewenstein et al. report that electrical and dye coupling is decreased with a [Ca^2+^]_i_ rise at junctional membranes [43]. Further evidence demonstrates that significantly lower [Ca^2+^]_i_, in the range of ~100 nM to low µM, can effectively regulate gap junction channel gating in salivary gland cells [44,45] and mammalian cardiac fibers [46]. In cultured chicken lens embryonic cells, cell–cell dye transfer is drastically reduced when exposed to Ca-ionophores A23187 or ionomycin, two compounds that increase [Ca^2+^]_i_ to ~110 to ~400 nM. Lowering the [Ca^2+^]_i_ to ~260 nM by saline containing EGTA results in cells ability to regain dye transfer capacity [47]. This indicates the role of Ca^2+^ as a modulator of cell communication via gap junction channels. Moreover, [Ca^2+^] is mediated by pH and affects gap junction gating [48]. *Xenopus* oocytes with Cx38 intracellularly buffered with BAPTA show that a low pH uncouples cells by increasing Ca^2+^ concentration [49]. Acidification to pH 6 has no effect on gating if Ca^2+^ is carefully buffered to resting levels by BAPTA in the patch pipettes [50].

Numerous studies support the involvement of CaM in the regulation of gap junction channels [51,52,53,54]. Cxs lack direct Ca^2+^ binding sites; however, a CaM biding site has been identified in the second half of the Cx cytoplasmic loop [55]. Moderate increase of [Ca^2+^]_i_ induces the activation of CaM-dependent kinases (CamK) [56]. Cxs are substrates of several kinases, which phosphorylate residues primarily at the C-terminal domains. This region of the connexins is the most diverse between Cxs. Phosphorylation regulates Cx channel/hemichannel function and protein turnover [57]. It has been reported that Cx32, Cx36, and Cx43 are substrates of CamKII [58,59,60]. Gap junction channels formed by Cx36 are more active after phosphorylation by CamKII [58,60]. CamKIIα has been detected in lens fiber membranes [61]. Mild [Ca^2+^]_i_ increase activates Cx45 gap junction communication. However, there is no evidence that CaMKII directly phosphorylates Cx45 [62]. The physiological effect of CamK on lens Cx channel/hemichannel remains elusive given the sequence diversity of Cx C-terminal domains. After a sustained increase of [Ca^2+^]_i_, cytoplasmic acidification, or Ca^2+^ ionophores are all shown to activate CaM, which interacts with the cytoplasmic loop domain of the Cxs, and leads to the reduction of Cx gap junction channel activity (chemical gating). CaM mutants lacking one or more of the four high-affinity Ca^2+^-binding sites strongly inhibit the formation of functional Cx32 channels [48]. Transient expression of the Cx43-EYFP mutant with deletion of the putative Cx43 CaM-binding site (residues 136–158 in the intracellular loop of Cx43) in HeLa cells eliminates the Ca^2+^-dependent inhibition of Cx43 gap junction permeability. These data suggest that the intracellular loop domain of Cx43 is the CaM-binding site that mediates the regulation of Cx43 gap junction channel permeability [54]. Our data also suggested that the interaction between Cx46 IL and CaM did not occur without Ca^2+^, whereas the dissociation constant between Cx46 IL and CaM quantified by isothermal titration calorimetry (ITC) is 2.95 ± 1.5 μM in the presence of Ca^2+^ [63]. The addition of calmidazolium, a small molecule CaM inhibitor, mimetic peptides of CaM binding sites present on Cx43, or CamKII, antagonize the inhibition of gap junction channel conductance induced by the increase of intracellular Ca^2+^ concentration [53]. Consistently, Cx44, the sheep ortholog of human Cx46, interacts with CaM in the presence of free Ca^2+^ and increases its intradomain cooperativity, thus enhancing its Ca^2+^-binding affinities by twofold [64]. Our data also suggested that the interaction between Cx46 IL and CaM did not occur without Ca^2+^, whereas the dissociation constant between Cx46 IL and CaM quantified by isothermal titration calorimetry (ITC) is 2.95 ± 1.5 μM in the presence of Ca^2+^ [59]. Gap junction communication mediated by Cx44 channels is reduced by the increase of intracellular Ca^2+^ levels. However, the pre-incubation with CaM antagonist prevents the uncoupling induced by the rise of cytoplasmic Ca^2+^. More recently, the missense mutation G143R located in the intracellular loop (IL) domain of Cx46 is identified in a four-generation Chinese family with congenital Coppock cataracts, which is also the only mutation identified in the IL domain region of Cx46 linked to congenital cataracts [65]. Interestingly, the G143 residue is located at a predicted CaM binding domain of Cx46 [63]. The opacity of this cataract is located between the nucleus and the posterior pole of the lens. Cx46 G143R lenses show larger junctional plaques at cell–cell interfaces, but decreasing gap junctional coupling in terms of the frequency of dye transfer in a dominant negative manner when compared to wild-type Cx46 expressing cells. The reduced gap junctional coupling is likely a result of non-conductivity of the channels [66].

## 4. New Mechanism of Ca^2+^/CaM in Regulation of Cx46 Hemichannels

Cx46 hemichannels are less-specific, big capacity channels with a preference to cations [67]. The unregulated opening of these hemichannels is not compatible with cell life. Cx46 hemichannel regulation is achieved by means of several mechanisms. As an example, treatment with the phorbol esters, protein kinase C (PKC) activators, reduces hemichannel currents, and inhibits channel activation (opening) [68,69]. It has been shown that casein kinase II participates in the stabilization of an open hemichannel state evoked by a 60 mV depolarizing pulse [70]. Cx46 hemichannels are also sensitive to nitric oxide and the nitrosylation of intracellular cysteines, leading to promotion of a faster activation rate, increased voltage sensitivity, and increased tail currents with altered kinetics [71]. Moreover, lipids, such as linoleic acid, an unsaturated fatty acid, have a biphasic effect on Cx46 hemichannel function, increasing hemichannel currents at 0.1 μM and reducing them at concentration over 100 μM. These effects are achieved without affecting gap junction channel activity [72]. One of the most common lipid peroxides products 4-hydroxynonenal (4-HNE) that can modify extracellular cysteines and reduce the activity of Cx46 hemichannels [73]. Cx hemichannels are highly active under low or an absence of extracellular Ca^2+^ conditions [8]. Cx46 hemichannel activity is regulated by voltage and the concentration of extracellular Ca^2+^. Hyperpolarization of the cell membrane and physiological extracellular Ca^2+^ concentrations keep Cx46 hemichannels inactive. We have recently demonstrated the increased hemichannel activity indicated by cellular uptake of positively charged ethidium bromide (Mw: 394 Da) in the presence and absence of extracellular in HeLa cells expressing the Cx46 G143R mutant [63]. This increased hemichannel activity is further confirmed by a negative charged fluorescence tracer dye, Alexa350 (Mw: 410 Da). These data support the notion that the increase of membrane permeability is not selective to charge. Additionally, the G143R mutation abolishes voltage-dependent ionic conductance in a dominant-negative manner in both *Xenopus* oocytes and HeLa cells. However, oocytes expressing Cx46 G143R show reduced membrane potential compared with those expressing WT Cx46. This suggests that the G143R mutation forms leaky hemichannels insensitive to voltage regulation. CaM has been shown to play a key role in regulating Cx46 hemichannel activity. The rate of dye uptake, a technique to visualize Cx hemichannels activity, is greatly reduced in the presence of the CaM inhibitor, calmidazolium chloride. Ionomycin, which raises the cytoplasmic level of Ca^2+^ by increasing influx of Ca^2+^ through the plasma membrane, leads to a dramatic increase in hemichannel activity observed in both WT and G143R mutants. The CaM inhibitor, however, greatly reduces the increased dye uptake induced by ionomycin. We point out that the increase in hemichannel activity is mediated by elevated intracellular Ca^2+^ and the activation of CaM. Although extracellular Ca^2+^ inhibits hemichannel opening, intracellular Ca^2+^ is reported to positively regulate Cx43 and Cx32 hemichannel activity [74,75].

**Figure 1:**
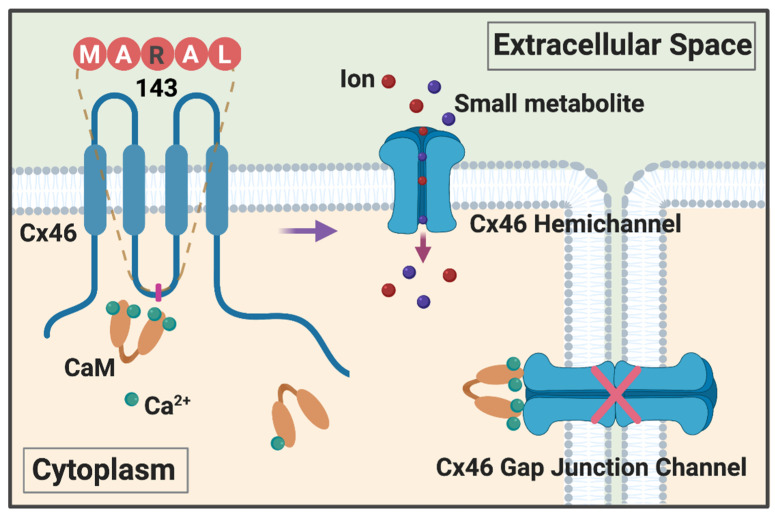
Illustration of molecular mechanisms of Ca^2+^-CaM regulation of Cx46 channel activity. The G143R mutation at the Cx46 intracellular domain alters its secondary structure, resulting in an increased interaction between Cx46 and the Ca^2+^-CaM complex, inducing a reduction of gap junction conductance and a decrease of sensitivity to Ca^2+^ and pH. At the same time, enhanced association of Cx46 with CaM decreases Cx46 hemichannel voltage sensitivity, but increases hemichannel permeability to small molecules. The presence of leaky hemichannels and lack of electrical and metabolic coupling is not compatible with lens fiber homeostasis, leading to cataract formation.

The underlying molecular mechanism is further illustrated more recently as shown in Figure 1. The α-helix of the connexin intracellular domain (IL) stabilizes its protein conformation and contributes to the CaM interaction [76]. We have determined the secondary structure of a GST-fusion protein containing the Cx46 IL domain of WT or G143R mutant using circular dichroism (CD). We find an altered alpha-helical structure of the Cx46 IL domain in response to pH and Ca^2+^. G143 is a highly conserved amino acid residue across members of the connexin family from various animal species. This mutation, in which the small hydrophobic glycine is replaced with the large, positively charged asparagine, results in a great reduction in α-helical structure of the IL domain. Previous studies report the co-localization of CaM and connexins [63,77,78,79], such as Cx35, as evidenced by immunostaining studies [80]. Our observation also corroborates the co-localization of Cx46 and CaM in HeLa cells in the presence of a high intracellular Ca^2+^ concentration. Under Ca^2+^ depletion conditions, this co-localization is compromised only in WT, but not in mutated Cx46-expressing cells. Moreover, the G143R mutation is located in the CaM-binding pocket in the IL domain. The G143R mutation enhances the interaction between the Cx46 IL domain and CaM as shown by a protein pull-down assay. Isothermal titration calorimetry (ITC) further shows a higher binding affinity between CaM and the G143R mutant IL with both high (2 mM) and low (0.3 mM) [Ca^2+^]. In the absence of Ca^2+^, no interaction between the WT IL domain and CaM is observed, whereas the interaction between CaM and the G143R mutant is still detectable. This lack of adaptability to the in situ physiological environment by the mutation might partially be responsible for malfunction of channel activities [63]. There are two possible explanations in terms of how Ca^2+^/CaM and connexin interactions regulate connexin channel function: one is that the interaction with CaM directly regulates gap junction channel gating, such as the reported mechanism for Cx32 [81]. Another mechanism is indirect, through the complex formation between Ca^2+^/CaM and connexin and activation of other signaling mechanisms, such as PKC or inositol 1,4,5-trisphosphate (IP_3_) [82]. Our studies with the G143R mutant implicate a direct regulation of connexin channels by CaM. The reduction of hemichannel function of the mutant by a CaM inhibitor could be due to disruption of the interaction between mutant Cx46 and CaM. The aberrant enhancement of the interaction by the G143R mutation with CaM and dysregulation of connexin hemichannels might contribute to homeostasis impairment and cataract formation in the lens.

## 5. Impacts of Lens Ca^2+^ Homeostasis and Its Dysregulation on Cx46 Channels and Vice Visa

Changes in membrane potential appear to play an essential role in hemichannel activity [15,16]. Under resting membrane potential, CaM activation by Ca^2+^ and binding to Cx46 are tightly regulated components of hemichannel function. Cx46 channels are known to play an essential role in maintaining lens homeostasis, and recently more evidence indicates that connexin hemichannels may also be involved in transferring reductants, like glutathione [25,83]. This suggests that the membrane leaky activity observed by Ebihara et al. could be required for the exchange of reductants from the extracellular space [84]. The lens microcirculation model indicates that gap junction communication is a vital component in excreting waste metabolites and the removal of Na^+^ and Ca^2+^ out of the lens fibers [16]. However, gap junction channels facilitate the movement of nutrients and reductants from metabolically active cortical fibers to inactive lens core fibers [21]. Lens fibers are very tolerant to free intracellular Ca^2+^; however, pathological conditions like oxidative stress, inflammatory signals, depolarization, hypoxia, and hypercalcemia increase the membrane permeability through Cx hemichannels allowing the efflux of metabolites, like ATP, NAD^+^ [20]. Permeable plasma membranes dissipate ionic gradients reducing the extracellular [Ca^2+^] and Na^+^. This change provokes depolarization and increase of [Ca^2+^]_i,_ leading to CaM activation, which stabilizes Cx46 hemichannels in a leaky conformation [63]. Concomitantly, active CaM decreases gap junction communication by binding Cx50 [85] and Cx46 channels and interrupts the microcirculation [66]. These events generate a vicious cycle that could ultimately lead to cataract formation. The G143R mutation with aberrant CaM binding enhances hemichannel activity even under normal physiological conditions and not under a depolarized state. The increased membrane leakiness due to dysregulated hemichannels leads to loss of voltage sensitivity, decreased cell viability, and the resistance of cells to oxidative stress [66]. Aberrant function of Cx46 hemichannels with the G143R mutation has the potential to disrupt lens cell homeostasis and consequently lead to cataract formation. Lens connexin mutations associated with cataracts are mainly located in the transmembrane and extracellular loop. There are an estimated 40 mutations of lens Cxs associated with cataracts [86]. The study of the Cx46-G143R mutation allows us to probe the regulatory mechanisms of connexin channels with regard to lens microcirculation, and the role hemichannels play in membrane permeability. Diametrically opposed regulation of gap junction and hemichannels has been observed in proinflammatory and oxidative stress conditions [87,88]. In both scenarios, the result is an increase of intracellular Ca^2+^ and the activation of multiple kinases [86,87]. However, the mechanism of opening of hemichannels induced by Ca^2+^ has been not been fully explored. Moreover, the proteins and the chain of events in this opposite regulation remain unknown. The role of CaM as common regulator of gap junction communication and hemichannels activity has not been investigated in detail. The evidence suggests that inhibitors of CaM may provide advantages that could keep the beneficial role of gap junction communication during injury, such as ion buffering [89,90]. Furthermore, these inhibitors favor a closed state of hemichannels, which can reduce the leakage of stress signals such us ATP and glutamate, known players that lead the secondary damage propagation [89,91].
